# Red-Haired People’s Altered Responsiveness to Pain, Analgesics, and Hypnotics: Myth or Fact?—A Narrative Review

**DOI:** 10.3390/jpm14060583

**Published:** 2024-05-29

**Authors:** Annelie Augustinsson, Elisabeth Franze, Martina Almqvist, Margareta Warrén Stomberg, Carina Sjöberg, Pether Jildenstål

**Affiliations:** 1 Care in High Technological Environments, Department of Health Sciences, Lund University, 221 00 Lund, Sweden; 2 Institute of Health and Care Sciences, Sahlgrenska Academy, University of Gothenburg, 405 30 Gothenburg, Sweden; 3 Department of Anesthesia and Intensive Care, Institute for Clinical Sciences, Sahlgrenska Academy, University of Gothenburg, 405 30 Gothenburg, Sweden; 4 Department of Anesthesiology, Surgery and Intensive Care, Sahlgrenska University Hospital, 405 83 Gothenburg, Sweden; 5 Department of Anesthesiology and Intensive Care, Örebro University Hospital and School of Medical Sciences, Örebro University, 701 85 Örebro, Sweden

**Keywords:** melanocortin-1 receptor, MC1R, redheads, pain, analgesics, hypnotics, response

## Abstract

Red hair has been linked to altered sensitivity to pain, analgesics, and hypnotics. This alteration may be impacted by variants in the melanocortin-1 receptor (*MC1R*) gene, which are mainly found in redheads. The aim of this narrative review was to explore and present the current state of knowledge on red hair and its plausible associations with altered responsiveness to pain, analgesics, and hypnotics. Structured searches in the PubMed, CINAHL Complete, and Scopus electronic databases were conducted. Evidence suggests that women with red hair have an increased sensitivity to pain. Conversely, data also indicate a higher pain tolerance in homozygous carriers of *MC1R* variant alleles. Varied responses to analgesia have been reported, with both increased analgesic responsiveness in homozygous carriers of *MC1R* variant alleles and less analgesia in redheads. Data indicate an increased need for hypnotics in redheads. However, failed attempts to find statistical associations between red hair and altered responsiveness to hypnotics are also evident. Even though there seems to be an association between red hair and an altered responsiveness to pain, analgesics, and/or hypnotics, the results of this narrative review are inconclusive. Further research studies with larger populations and *MC1R* testing are needed.

## 1. Introduction

There is uncertainty regarding redheads’ responsiveness to pain, analgesics, and hypnotics. Redheads’ plausible altered responsiveness seems to be associated with a dysfunction in the melanocortin-1 receptor (MC1R), a receptor that besides contributing to red hair also contributes to a more sensitive light skin with poor tanning ability, predisposition to sunburn, and increased risk for melanoma [1,2]. MC1R is a seven-transmembrane G-protein-coupled receptor primarily located on the surface of melanocytes and transformed melanoma cells. The receptor is activated by melanocyte-stimulating hormone (MSH) and/or adrenocorticotropic hormone (ACTH), controlling melanogenesis [3]. The receptor plays a crucial role in determining human pigmentation by regulating which type of melanin, i.e., the dark brown/black UV protective eumelanin or the red/blonde pheomelanin pigment, will be produced [3,4]. Individuals with a dysfunctional MC1R may therefore have a decreased synthesis of eumelanin, which leads to fair skin and an increased sensitivity to UV exposure.

**Figure 1:**
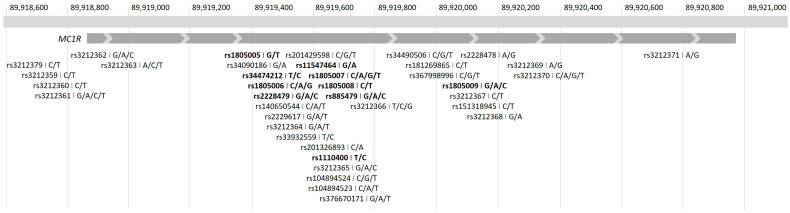
The genomic region of *MC1R* at chromosome 16 and *MC1R* SNPs. SNPs associated with red hair are highlighted with bold text.

MC1R is encoded by the highly polymorphic *MC1R* gene, which consists of a single exon and is located on chromosome 16. Common variants, i.e., single-nucleotide polymorphisms (SNPs), in the *MC1R* gene are associated with normal differences in skin and hair color, providing evidence of its association with normal human pigment variation (Figure 1) [5,6]. Genetic loss-of-function variants in *MC1R* appear mostly in red-haired individuals [3], both in homozygous and compound heterozygous carriers of the variant alleles [7].

Globally, the distribution of *MC1R* polymorphisms varies widely across different regions. People with altered function of the MC1R are mostly found in Northern Europe, although carriers of *MC1R* variants are also found among red-haired European descendants in South Africa and Australia as well as in darker-skinned Southern Europeans, Mongolians, and Jamaicans [4]. 

The aim of this narrative review was to explore and present the current state of knowledge on redheads’ responsiveness to perceived pain, analgesics, and hypnotics.

## 2. Materials and Methods

**Figure 2:**
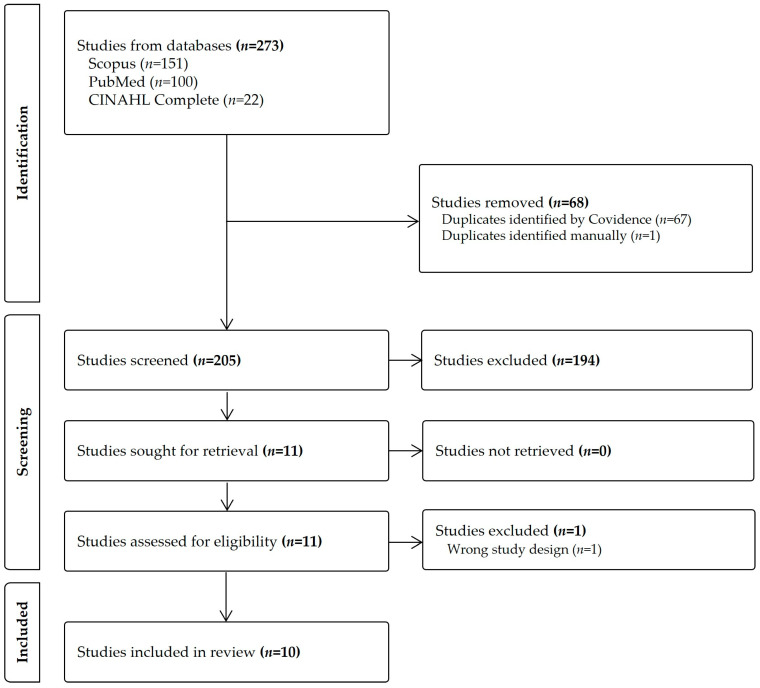
A PRISMA flowchart of study identification, screening, and inclusion.

Advanced literature searches in the PubMed, CINAHL Complete, and Scopus electronic databases were performed to identify studies investigating redheads’ responses to pain, analgesics, and/or hypnotics. The following search strategy was used: ((“red hair” OR redhead OR red-haired OR “melanocortin-1 receptor” OR MC1R) AND (pain OR analgesics OR anesthetics OR “general anesthesia” OR hypnotics OR sedative OR anaesthesia OR anesthesia)). Randomized controlled trials (RCTs), clinical trials, and observational studies regarding adult (≥18 years of age) red-haired individuals’ responses to pain, analgesics, and/or hypnotics were included. No restriction regarding the publication date was set. The search strategy identified potential studies for the narrative review. These were subsequently uploaded to Covidence for the PRISMA-based screening and selection process (Figure 2). The grading of recommendations, assessment, development, and evaluations (GRADE) approach was used to grade the quality of evidence [8] and the Critical Appraisal Skills Programme (CASP) was used to check the trustworthiness, results, and relevance of the articles [9]. Studies that did not meet the aim of the study or were not of high or medium quality were excluded. A total of ten original studies were included in the final analysis, of which eight were conducted between 2003 and 2013 and only two were conducted in the last ten years. They consisted of three observational studies (one of which had extracted retrospective data for a secondary analysis) and seven case-control studies. Across these 10 studies, sample sizes ranged from 20 to a total of 32,174 participants. The median sample size was 45 participants. Five studies specifically evaluated the association between red hair and the responsiveness to pain or to both pain and analgesics. The other five studies evaluated the effect of hair color on responsiveness to hypnotics. Key characteristics of the ten included studies were extracted and are summarized in Table 1. Reported associations between red hair and responsiveness to pain, analgesics, and/or hypnotics across the included studies are presented in Table 2.

## 3. Results

### 3.1. Altered Responsiveness to Pain

Evidence suggests that women with red hair have an increased sensitivity to pain. In a study by Fontanillas et al. [19], the relationship between the human genome and pain sensitivity, specifically regarding hair color and the *MC1R* gene, was evaluated. Here, a total of 25,321 participants answered a pain sensitivity questionnaire (PSQ) and 6853 participants performed a cold pressure test (CPT). The results showed that red-haired women had higher PSQ scores compared to women with other hair colors and men (*p* = 0.046). However, no significant difference could be detected between red-haired men and men with other hair colors.

Based on the hypothesis that red-haired women are more sensitive to pain, Liem et al. [13] evaluated differences between redheads (*n* = 30) and non-redheads (*n* = 30). In this study, participants’ responsiveness to pain was tested with the Neurometer^®^ CPT/C and the TSA-II NeuroSensory Analyzer device. Results showed that women with red hair were more sensitive to both cold and heat stimuli (*p* = 0.001 and *p* = 0.009, respectively) compared to non-redheads. Conversely, no significant differences regarding responses to pressure- and heat-induced pain between red-haired women (*n* = 20) and women with other hair colors (*n* = 20) were reported in a study by Andresen et al. [15], where responses to pain through heat and pressure stimulations and topical application of capsaicin were evaluated. However, this study indicated that redheads were significantly less sensitive to capsaicin-induced hyperalgesia compared to non-redheads (*p* = 0.014). 

Both Liem et al. [13] and Andresen et al. [15] examined pain response to heat. The studies were conducted in a similar way and on similar populations. Even though Liem et al. showed a significant difference in the response to thermal stimuli in red-haired women compared to women with other hair colors [13], Andresen et al. reported no difference between the groups [15]. These results could possibly be explained by the use of different temperatures in the studies, which may have resulted in varied nociceptive responses [21].

In addition, studies on individuals who were genotyped for *MC1R* variants showed varied results on pain responsiveness between *MC1R* variant carriers and non-carriers. In the study by Fontanillas et al. [19], significantly higher PSQ scores were reported by carriers of one (*p* = 0.0068) or two or more (*p* = 0.015) *MC1R* SNPs compared to carriers of wild-type alleles. Conversely, they did not observe an association between being a carrier of *MC1R* variants and CPT duration. However, the tests in this study were conducted in the participants’ home environments without the presence of a researcher. Therefore, it is challenging to ascertain whether they were performed correctly. This could have led to compromised reliability of the investigation and, consequently, varied validity of the results. In contrast, in a study by Mogil et al. [14], individuals with *MC1R* variants exhibited significantly higher pain tolerance compared to controls when acute pain was induced through electrodes placed on the skin over the tibial bone of the left leg (*p* = 0.018).

### 3.2. Altered Responsiveness to Analgesics

In another study by Mogil et al. [10], the responsiveness to analgesics after being given ischemic and thermal pain stimuli was evaluated in 42 participants who underwent genotyping for *MC1R* variants. *MC1R* variants were shown to significantly influence the response to analgesia, however, only in red-haired women and not in red-haired men. When using the κ-opioid Pentazocine, women with two *MC1R* variant alleles were the only ones that had a clear effect of, and response to, the given analgesics. In the more recent study published by the same authors [14], it was further investigated whether the difference in response to analgesics depended on the received type of opioid. The results indicated that *MC1R* variant carriers had an increased response to µ-opioid (morphine) induced analgesia compared to the control group (*p* = 0.003). Whereas the first study by Mogil et al. [10] showed a difference in the responsiveness to analgesics between women and men, which is consistent with results from other previous studies, i.e., that gender has an association with perceived pain [22,23], no significant difference between sexes could be demonstrated in the more recent study [14]. Further investigation is needed to determine whether the observed difference in response is attributable to gender in general or if it is directly linked to μ- and κ-opioids.

The responsiveness to analgesia in redheads and non-redheads was also evaluated in the study by Liem et al. [13]. When the effect of the subcutaneously administered lidocaine was evaluated, a lower effect was shown in redheads compared to dark-haired participants, where redheads showed significantly lower pain tolerance thresholds at 2000 Hz, 250 Hz, and 5 Hz stimulation (*p* = 0.005, *p* = 0.03, and *p* = 0.013, respectively). However, no significant difference was observed between the two groups in response to cutaneous administered lidocaine when evaluating the effect of local anesthetics (*p* = 0.46). While both studies by Mogil et al. [10,14] showed an increased response to opioids in *MC1R* SNP carriers compared to the controls, Liem et al. [13] showed that red-haired women had a weaker response to analgesics. This may be explained by the variation in pain character, the use of different types of analgesics, and different ways of administering the drug [24]. Another possibility is that the individual’s own experience of pain could have affected the outcome, i.e., perceived pain, in both tests, even though participants who had been part of earlier pain studies were excluded [14]. It is difficult to draw any conclusion from the studies by Mogil et al. [10,14] and Liem et al. [13]. These studies included small populations and the researchers were not blinded to participants’ hair color. In addition, it must be considered that the researchers could have influenced the results by observational bias, and participants could have modified their behavior knowing that they were part of a study, the so-called Hawthorne effect [20]. The participants had, however, not been informed of the hypotheses of the studies, which may strengthen the results. 

### 3.3. Altered Responsiveness to Hypnotics

Redheads may require more sedation compared to non-redheads. Studies regarding a possible increased need for hypnotics in redheads measured the used amount of volatile anesthetic agent and hypnotic depth, e.g., using the bispectral index (BIS) and end-tidal inhalation concentration, i.e., minimal alveolar concentration (MAC). In an experimental study by Liem et al. [12], all participants (*n* = 20) underwent genotyping for *MC1R* variants. Study participants were anesthetized by sevoflurane and subsequently transitioned to desflurane, maintaining anesthesia with an end-tidal concentration of 5.5–7.5%. The response to anesthesia was measured by bilateral electrical stimuli on the front of the thighs for ten seconds using transdermal needles. If the person moved their legs in response to the stimuli, the amount of volatile agent was increased by 0.5%. In contrast, if there was no response, the amount was decreased by 0.5%. The study showed a significant difference in the need for volatile agents between red-haired women and the control group, with redheads requiring 19% more (*p* = 0.0004). Additionally, Chua et al. [11] conducted a blinded randomized study on redheads’ responsiveness to hypnotics, in which the participants (*n* = 39) received either intravenous midazolam or saline. The study showed significant differences between the groups regarding the observer’s assessment of alertness/sedation (OAA/S) and the drowsiness visual analog scale (VAS), where the redheads required more sedation compared to non-redheads (*p* = 0.004 and *p* = 0.034, respectively). BIS values were not different between the groups [12]. No significant differences between redheads and non-redheads in terms of anesthetic requirements were reported in the three studies by Myles et al. [16], Gradwohl et al. [18], and Doufas et al. [17], where none of the participants were genotyped for *MC1R* variants. The study by Gradwohl et al. [18] was conducted on a large population. However, it included patients at higher risk of intraoperative awareness, resulting in them receiving larger doses of both volatile and intravenous anesthesia as well as muscle relaxants. Because they were given higher doses of medication, it may have affected the result [25].

To measure responses to hypnotics, most of the studies used the BIS tool [11,16,17,18]. Only Chua et al. [11] used several different validated measuring tools. It is interesting, though, that the only objective measuring tool where one can imagine that the evidence is more reliable, no significant differences were shown, neither in the study by Chua et al. [11] nor in any of the other studies [16,17,18]. This raises the question of whether the results would have been different if other objective measuring tools had been used. Even Gradwohl et al. [18] question the use of BIS to assess the depth of anesthesia as it is a surrogate measure and does not fully reflect the neurobiological depth of anesthesia. Hence, it is possible that redheads may have needed more volatile anesthesia than indicated. To obtain more valid results, consideration should have been given to the type of anesthesia each patient received. The measurement shown by electroencephalogram (EEG) monitoring varies according to the type of anesthesia the patient receives and the patient’s age can also affect the results [26]. The studies that evaluated the response of hypnotics took place between 2004 and 2015. It is possible that the outcome would have been different if the studies had been implemented in the present due to the progress of medical techniques. In addition, there is a possibility that the results could have been different if another measuring tool, e.g., spectral edge frequency (SEF), had been used as well since it shows more individualized EEGs with higher precision [26].

Only one of the studies where the participants were genotyped for *MC1R* variants evaluated the responsiveness to hypnotics. In this study, by Liem et al. [12], a significant increase in the requirement of volatile anesthesia in redheads compared to the controls was reported. However, the MAC value was lower than what is usually needed for surgical stimuli in this study. This was expected though since the depth of anesthesia should always be adjusted according to the patient’s age and the anticipated stimuli [27]. In the more recent study by Liem et al. [13], one could also assume that all red-haired participants had two *MC1R* variant alleles since the genetic analysis from the earlier study had evidence for this [20]. This also coheres with other studies that have examined genetic *MC1R* SNPs associated with red hair [28,29]. However, Mogil et al. [10] reported that individuals with red hair can have a functioning MC1R and that the red hair color could be caused by something else than a non-functioning MC1R. With regard to the hypothesis that variants in *MC1R* affect the responsiveness to pain, analgesics, and hypnotics, it is possible that the outcome could have been different in the studies where the participants were not genotyped. In addition, it is difficult to know if any exclusion criteria were made with regard to the patients’ habits, e.g., use of drugs and/or alcohol, which could have increased the need for hypnotics [30].

Variants in the *MC1R* gene are mostly known for their regulation of pigmentation of skin and hair color [6] but may also be associated with pain sensitivity and altered responsiveness to analgesics and hypnotics. This could indicate that genetic variations in *MC1R* result in altered responsiveness and would therefore be of clinical relevance [1,31].

## 4. Conclusions

The largest challenge for people with red hair seems to be an altered responsiveness to pain and analgesics. It is important to implement multimodal pain management since redheads have shown varied sensitivity to opioids. In addition, redheads seem to have a certain tolerance toward volatile anesthesia, which leads to total intravenous anesthesia being preferred. EEG-based depth of anesthesia monitoring should be used to increase patient safety. One consideration for future preoperative routines regarding this population of individuals might be to enhance the possibility of genetic testing of *MC1R*. It seems that studies regarding the responsiveness to pain, analgesics, or hypnotics in red-haired people are limited. The results of this narrative review indicate a need for more randomized intervention studies. Few studies have been made, and mostly on relatively small populations, with a scarcity of studies incorporating *MC1R* genotyping. Due to population heterogeneity, certain genotypes may hold importance in determining the effects of medication. Hence, pharmacogenomic associations need validation for each therapeutic indication, particularly with the advent of new drugs and in diverse subgroups. Recognizing these limitations is crucial for accurately interpreting genetic factors influencing drug response and for effectively translating pharmacogenomic findings into clinical practice. Because the studies had different designs and populations, it was difficult to draw any general conclusions. However, this narrative review shows some associations between red hair, including people with genetic variants in *MC1R*, and altered responsiveness to pain, analgesics, and hypnotics. Further studies on this subject are, however, needed, including larger populations and *MC1R* genotyping.

## Figures and Tables

**Table 1:** Key characteristics of the ten included studies, arranged by year of publication.

Authors	Study Design	Sample Size (*n*)		Primary Objective	Intervention	Analysis of *MC1R*	Quality
		Total	Red-Heads				
Mogil et al. (2003) [10]	Experimental case-control study	42	21	To evaluate redheads’ and non-redheads’ responses to κ-opioids and pain.	Thermal and ischemic pain assessments after administration of κ-opioids or saline.	Yes	High
Chua et al. (2004) [11]	Experimental case-control study	39	20	To evaluate resistance to sedative drugs in redheads and non-redheads.	Pain assessment after experimentally induced pain through application of capsaicin.	No	High
Liem et al. (2004) [12]	Experimental case-control study	20	10	To evaluate whether women with red hair have a greater requirement of volatile anesthesia than women with dark hair.	Measurements of depth of anesthesia through pain stimuli with bilateral intradermal needles in the anterior thighs and end-tidal concentration of inhaled gas.	Yes	High
Liem et al. (2005) [13]	Experimental case-control study	60	30	To evaluate if women with red hair are more sensitive to pain compared to women with dark hair.	Pain assessments through cold and heat stimuli during local anesthesia, both subcutaneous and on the skin.	No	High
Mogil et al. (2005) [14]	Experimental case-control study	47	29	To evaluate redheads’ responses to pain and µ-opioid analgesia compared to individuals with other hair colors.	Assessments of electrode-induced acute pain in the legs after administration of µ-opioids.	Yes	High
Andresen at al. (2010) [15]	Experimental case-control study	40	20	To evaluate if pain sensitivity differs between women with red hair compared to women with blond/dark hair.	Pain assessments to different phasic experimental stimulus modalities and capsaicin-induced hyperalgesia.	No	High
Myles et al. (2012) [16]	Prospective, matched cohort study	468	32	To evaluate the effects of hair color on requirements and response to general anesthesia and on postoperative recovery.	Measurements of end-tidal concentration and depth of volatile anesthesia.	No	Moderate
Doufas et al. (2013) [17]	Experimental case-control study	29	13	To evaluate the hypnotic effect of propofol in healthy volunteers with red and dark hair.	Measurements of BIS and drug concentrations before, during, and after anesthesia.	No	High
Gradwohl et al. (2015) [18]	Prospective cohort study	1914	319	To evaluate the risk of awareness, need for anesthesia, and postoperative recovery in redheads.	A secondary analysis where redheads were compared to non-redheads.	No	Moderate
Fontanillas et al. (2022) [19]	Cohort study	PSQ: 25,321 CPT: 6853	PSQ: 693 CPT: 240	To identify genetic factors contributing to individual pain perception.	Self-assessment forms and CPT intervention in the participants’ home environments.	Yes	High

Abbreviations: BIS, bispectral index; CPT, cold pressor test; MAC, minimum alveolar concentration; *MC1R*, melanocortin-1 receptor; PSQ, pain sensitivity questionnaire.

**Table 2:** Reported associations between hair color and responsiveness to pain, analgesics, and/or hypnotics in the ten included studies, arranged by year of publication.

Authors	Response to	Measures	Hair Color or *MC1R* Genotype			Comments
			Redheads or	Non-Redheads or 0/1 Variant Allele	*p* Value	
			>1 Variant Alleles			
Mogil et al. (2003) [10]	Pain and analgesics	IPT:				Significantly greater pentazocine analgesia in women with >1 *MC1R* variant alleles than in women with 0/1 allele, and significantly higher ischemic PT and ratings of intensity and unpleasantness in women with >1 *MC1R* variant alleles than in women with 0/1 allele, but not in men.
		Women	408.0 (284.1)	37.1 (82.9)	<0.05	
		Men	46.4 (149.3)	84.1 (182.8)	>0.05	
		SIPI:				
		Women	84.8 (50.3)	16.9 (41.9)	<0.05	
		Men	18.7 (36.2)	37.8 (38.3)	>0.05	
		SIPU:				
		Women	79.6 (45.7)	9.0 (49.6)	<0.05	
		Men	24.2 (41.0)	33.0 (49.0)	>0.05	
		STPI:				
		Women	28.0 (48.9)	–22.8 (40.1)	<0.05	
		Men	9.2 (51.0)	26.2 (56.5)	>0.05	
Chua et al. (2004) [11]	Hypnotics	OAA/S:				Significantly less sedation and cognitive impairment in volunteers with red hair than in those with blond or brown hair. BIS values did not differ between groups.
		Placebo	5.0 (0.0)	5.0 (0.0)	>0.05	
		Midozolam	5.0 (0.0)	4.0 (2.0)	0.004	
		Drowsiness VAS:				
		Placebo	0 (5)	0 (3)	>0.05	
		Midozolam	42 (54)	67 (47)	0.034	
		BIS:				
		Placebo	97 (3)	97 (4)	>0.05	
		Midazolam	85 (13)	80 (9)	>0.05	
Liem et al. (2004) [12]	Hypnotics	Desfluran requirement (%)	6.2 (5.9–6.5)	5.2 (4.9–5.5)	0.0004	A significant increase in volatile anesthesia requirement in redheads compared with controls.
Liem et al. (2005) [13]	Pain and analgesics	CSPT (°C)	30.7 (30.3–31.0)	30.5 (29.9–31.2)	0.596	Significantly higher sensitivity to cold pain perception and tolerance, as well as lower PT threshold at stimulation after subcutaneous lidocaine in women with red hair than in women with dark hair.
		CPPT (°C)	22.6 (15.1–26.1)	12.6 (0.0–20.0)	0.004	
		CPTT (°C)	6.0 (0.0–9.7)	0.0 (0.0–2.0)	0.001	
		HSPT (°C)	33.8 (33.5–34.0)	33.5 (33.4–33.8)	0.015	
		HPPT (°C)	41.4 (39.7–43.1)	42.4 (41.3–44.6)	0.059	
		HSTT (°C)	46.3 (45.7–47.5)	47.7 (46.6–48.7)	0.009	
		PT thresholds (mA):				
		
		2000 Hz	11.0 (8.5–16.5)	>20 (14.5–>20)	0.005	
		250 Hz	5.0 (4.0–9.0)	11.6 (8.0–13.0)	0.003	
		5 Hz	6.3 (3.2–10.2)	8.5 (7.0–14.2)	0.013	
Mogil et al. (2005) [14]	Pain and analgesics	PT (mA):				Significantly greater PT and analgesic response in individuals with *MC1R* variants than in controls.
		Baseline	20.9 (1.7)	15.8 (1.2)	0.018	
		Ischemic	1.18 (0.04)	1.49 (0.09)	0.003	
Andresen at al. (2010) [15]	Pain	PTT (kPa/s)	713.6 (403.8)	673.5 (226.1)	0.80	No differences in PTT or HTT between redheads and controls.
		HTT (°C)	47.4 (2.8)	47.8 (1.7)	1.0	
Myles et al. (2012) [16]	Hypnotics	aaMAC	1.28 (0.27)	1.31 (0.34)	0.46	No evidence of increased anesthetic requirements in redheads compared with patients with black hair.
		Equivalents (mg × kg^−1^):				
		Midazolam	0.028 (9.026)	0.028 (0.026)		
		Morphine	0.80 (1.15)	0.75 (0.85)		
		Propofol	2.11 (2.00)	2.22 (1.75)		
		Vecuronium	0.12 (0.11)	0.12 (0.09)		
Doufas et al. (2013) [17]	Hypnotics	Ce_50 BIS_ (µg/mL)	2.57 (1.68–3.60)	2.71 (2.28–3.36)	>0.05	No difference in the hypnotic effect of propofol between volunteers with red and dark hair.
Gradwohl et al. (2015) [18]	Hypnotics	aaMAC	0.92 (1.18)	0.91 (0.18)	0.476	No difference in the anesthetic management between redheads and matched controls without red hair.
Fontanillas et al. (2022) [20]	Pain	Redhead × sex *	0.2597 (0.1300)		0.046	Significantly higher sensitivity to pain in red-haired women and self-perceived pain in individuals with >1 *MC1R* variant alleles.
		CPT: *				
		>1 alleles	0.1253 (0.0515)		0.015	
		0/1 allele	0.0517 (0.0191)		0.0068	

* Linear models on PSQ score. Abbreviations: aaMAC, age-adjusted minimum alveolar concentration; BIS, bispectral index; CPPT, cold pain perception threshold; CPT, cold pressor test; CPTT, cold pain tolerance threshold; CSPT, cold sensory perception threshold; HPPT, heat pain perception threshold; HPTT; heat pain tolerance threshold; HSPT, heat sensory perception threshold; HTT, heat tolerance threshold; Hz, Hertz; IPT, ischemic pain tolerance; MC1R, melanocortin-1 receptor; OAA/S, observer’s assessment of alertness/sedation; PSQ, pain sensitivity questionnaire; PT, pain tolerance; PTT, pressure tolerance threshold; SIPI, sum ischemic pain intensity; SIPU, sum ischemic pain unpleasantness; STPI, sum thermal pain intensity; VAS, visual analog scale.

## Data Availability

No new data were created.
